# Dissemination of macrolides, fusidic acid and mupirocin resistance among *Staphylococcus aureus* clinical isolates

**DOI:** 10.18632/oncotarget.19491

**Published:** 2017-07-22

**Authors:** Xingmei Liu, Shanshan Deng, Jinwei Huang, Yaling Huang, Yu Zhang, Qin Yan, Yanhong Wang, Yanyue Li, Chengfu Sun, Xu Jia

**Affiliations:** ^1^ Non-Coding RNA and Drug Discovery Key Laboratory of Sichuan Province, Chengdu Medical College, Chengdu 610500, China; ^2^ School of Laboratory Medicine, Chengdu Medical College, Chengdu 610500, China; ^3^ Institute of Antibiotics, The Fifth Affiliated Hospital, Wenzhou Medical University, Lishui 323000, China

**Keywords:** *Staphylococcus aureus*, macrolides, fusidic acid (FA), mupirocin, resistance

## Abstract

As an increasingly common cause of skin infections worldwide, the prevalence of antibiotic-resistant *Staphylococcus aureus* (*S. aureus*) across China has not been well documented. This literature aims to study the resistance profile to commonly used antibiotics, including macrolides, fusidic acid (FA) and mupirocin, and its relationship to the genetic typing in 34 *S. aureus* strains, including 6 methicillin-resistant *S. aureus* (MRSA), isolated from a Chinese hospital. The MIC results showed 27 (79.4%), 1 (2.9%) and 6 (17.6%) isolates were resistant to macrolides, FA and mupirocin, respectively. Among 27 macrolide-resistant *S. aureus* isolates, 5 (18.5%) were also resistant to mupirocin and 1 (3.7%) to FA. A total of 13 available resistant genes were analyzed in 28 antibiotic-resistant strains using polymerase chain reaction (PCR). The positive rates of macrolide-resistant *ermA*, *ermB*, *ermC*, *erm33* and low level mupirocin-resistant *ileS* mutations were 11.1%, 25.9%, 51.9%, 7.4% and 100%, respectively. Other determinants for FA- and high level mupirocin-resistance were not found. The results of multilocus sequence typing (MLST) and pulsed field gel electrophoresis (PFGE) revealed 13 sequence types (STs) and 18 clusters in 23 resistant gene positive *S. aureus* isolates. Among these STs, ST5 was most prevalent, accounting for 18.2%. Notably, various clusters were found with similar resistance phenotype and genotype, exhibiting a weak genetic relatedness and high genetic heterogeneities. In conclusion, macrolides, especially erythromycin, are not appropriate to treat skin infections caused by *S. aureus*, and more effective measures are required to reduce the dissemination of macrolides, FA and mupirocin resistance of the pathogen.

## INTRODUCTION

As one of the most common pathogens, *S. aureus* usually caused systemic and pyogenic local infections in both community settings and hospitals. Moreover, due to the existence of virulence factors of *S. aureus*, it tends to cause more widespread infections, such as meningitis, endocarditis and blood stream infections [1]. The topical antimicrobial agents, especially macrolide antibiotic erythromycin, fusidic acid (FA), mupirocin, are commonly used to treat skin infections caused by *S. aureus*.

Macrolides are widely used to treat acute upper and lower respiratory tract infections, sexually transmitted diseases and chronic pulmonary infections. In addition, they also applied to skin and soft tissue infections [2]. As the first macrolide antibiotic discovered in 1952, erythromycin played an important role in treating infectious diseases. Since then, more active semi-synthetic derivatives, such as azithromycin and clarithromycin, have been developed [3]. Resistance to macrolides gradually emerged along with their extensive use for treating *S. aureus*. So far, three kinds of mechanisms are responsible for resistance to macrolides in *S. aureus*, including an active efflux pump encoded by *msrA* gene, enzymatic inactivation of antibiotics, and ribosomal target modification in *ermA*, *ermB*, *ermC* and *erm33* genes. Among these three mechanisms, the last one is the primary mechanism involved [2, 4].

As an effective antibiotic, FA is often used to treat diseases, including skin and soft tissue infections, acute osteomyelitis, septic arthritis and other device related infections, which are caused by *S. aureus* [5]. Being extracted from cultures of *fusidium coccineum*, FA inhibits bacterial protein synthesis by preventing the turnover of *fusA* encoded elongation factor G (EF-G) from the ribosome [6, 7]. Point mutations in this chromosomal gene usually lead to the high level resistance of FA [8]. Low level resistance arises via the FusB-family proteins (encoded by *fusB, fusC* or *fusD*) which can protect drug target site from binding with FA molecules [9]. In addition, mutations in *rplF* encoded ribosomal protein L6 (collectively called fusE mutants) can also lead to low level FA resistance [10].

Mupirocin, also called pseudomonic acid A, is mainly used to treat skin, soft tissue and postoperative wound infections and can also eliminate nasal MRSA carriage from healthcare workers [11, 12]. As an isoleucine analogue produced by *Pseudomonas fluorescens*, mupirocin kills bacteria by competitively binding to isoleucine-tRNA synthetase (IRS), and consequently interfering with protein synthesis [13]. There are two categories of mupirocin resistances: low level resistance and high level resistance. Low level resistance is usually caused by point mutations, including A637G, G1762T, G1891T, T1984A and A2412T in the chromosomal staphylococcal isoleucine-tRNA synthetase (*ileS*) gene [14, 15]. High level resistance is commonly mediated by *mupA* (also referred to as *ileS2*) encoding an additional modified IRS [16, 17]. In addition, high level mupirocin resistance can also be caused by the mobile resistance gene *mupB* [18].

Antibiotic resistance to erythromycin, FA and mupirocin is an increasingly serious problem worldwide [19]. To provide an update to the antibiotic resistance of *S. aureus* in China, here, we systematically investigated the epidemiology and molecular characteristics of *S. aureus* from a university hospital in East China, and performed a comprehensive evaluation and comparison of their genetic diversity.

## RESULTS

### Antimicrobial susceptibility of *S. aureus* clinical isolates

To understand the resistance to macrolides, FA and mupirocin of 34 *S. aureus* isolates collected from a hospital, antimicrobial susceptibility testing was performed. The results showed that the resistance rates to erythromycin, clarithromycin, azithromycin, dirithromycin, FA and mupirocin accounted for 73.5% (25/34), 70.6% (24/34), 64.7% (22/34), 73.5% (25/34), 2.9% (1/34) and 17.6% (6/34), respectively (Table 1). The high resistance to macrolides in the present study indicated these macrolides were not applicable to treat *S. aureus* infections induced by resistant strains. Meanwhile, low resistance to FA and mupirocin indicated these two antibiotics remained effective for the therapy of infections caused by *S. aureus*. In total, we identified 28 antibiotic resistant isolates including 27 macrolide-resistant, 1 FA-resistant and 6 mupirocin-resistant isolates (Table 2).

**Table 1:** The antimicrobial resistance rates of *S.aureus* isolates including MSSA and MRSA

Antimicrobial agent	SA, % (n/34)	MSSA, % (n/28)	MRSA, % (n/6)
ERY	73.5% (25/34)	67.9% (19/28)	100.0% (6/6)
CLR	70.6% (24/34)	67.9% (19/28)	83.3% (5/6)
AZM	64.7% (22/34)	60.7% (17/28)	83.3% (5/6)
DTM	73.5% (25/34)	71.4% (20/28)	83.3% (5/6)
FA	2.9% (1/34)	3.6% (1/28)	0% (0/6)
MUP	17.6% (6/34)	21.4% (6/28)	0% (0/6)

ERY, erythromycin; CLR, clarithromycin; AZM, azithromycin; DTM, dirithromycin; FA, fusidic acid; MUP, mupirocin; SA, *Staphylococcus aureus*, MSSA, methicillin-susceptible *Staphylococcus aureus*; MRSA, methicillin-resistant *Staphylococcus aureus*.

**Table 2:** Molecular resistance characteristics of 28 antibiotic-resistant *S. aureus* isolates

Resistance phenotype (MIC in μg/ml)							Genotype					MRSA	MSSA
Isolate no.	Macrolides				FA	MUP	Macrolide-resistant gene				MUP-resistant gene mutaion		
	ERY	AZM	CLR	DTM			*ermA*	*ermB*	*ermC*	*erm33*	*ileS* A637G mutation		
016	256^R^	4^S^	256^R^	256^R^	0.125^S^	4^S^	-	-	-	-	-	-	+
017	256^R^	256^R^	256^R^	256^R^	0.125^S^	512^H^	-	-	+	-	+	-	+
022	256^R^	256^R^	32^R^	128^R^	0.125^S^	2^S^	-	+	-	-	-	-	+
023	4^S^	0.125^S^	0.25^S^	256^R^	0.125^S^	2^S^	-	-	-	-	-	-	+
026	256^R^	256^R^	256^R^	256^R^	0.125^S^	4^S^	-	-	+	-	-	-	+
029	256^R^	4^S^	256^R^	256^R^	0.125^S^	512^H^	-	+	-	-	+	-	+
034	256^R^	0.125^S^	0.25^S^	0.5^S^	0.125^S^	4^S^	-	+	-	-	-	-	+
038	256^R^	256^R^	256^R^	256^R^	0.125^S^	256^L^	-	+	+	-	+	-	+
041	256^R^	256^R^	256^R^	256^R^	2^L^	2^S^	-	+	-	-	-	-	+
049	2^S^	2^S^	0.25^S^	0.5^S^	0.125^S^	1024^H^	-	-	-	-	+	-	+
052	256^R^	128^R^	256^R^	256^R^	0.125^S^	4^S^	-	-	+	-	-	-	+
053	256^R^	128^R^	256^R^	>256^R^	0.125^S^	4^S^	-	+	-	-	-	-	+
055	64^R^	128^R^	256^R^	256^R^	0.125^S^	2^S^	-	-	+	-	-	-	+
060	256^R^	64^R^	256^R^	256^R^	0.125^S^	2^S^	-	-	+	-	-	-	+
083	256^R^	64^R^	256^R^	128^R^	0.125^S^	2^S^	-	-	-	-	-	-	+
085	256^R^	64^R^	256^R^	128^R^	0.125^S^	1024^H^	-	+	-	-	+	-	+
092	256^R^	64^R^	256^R^	128^R^	0.125^S^	2^S^	-	-	-	-	-	-	+
097	1^S^	64^R^	256^R^	128^R^	0.125^S^	1024^H^	+	-	-	-	+	-	+
107	256^R^	128^R^	256^R^	128^R^	0.125^S^	2^S^	-	-	+	-	-	-	+
108	256^R^	128^R^	256^R^	256^R^	0.125^S^	2^S^	+	-	+	-	-	-	+
114	256^R^	128^R^	256^R^	128^R^	0.125^S^	2^S^	-	-	+	-	-	-	+
116	256^R^	128^R^	256^R^	256^R^	0.125^S^	2^S^	-	-	+	-	-	-	+
117	64^R^	128^R^	256^R^	256^R^	0.125^S^	2^S^	-	-	+	-	-	+	^-^
118	64^R^	128^R^	256^R^	256^R^	0.125^S^	2^S^	+	-	-	+	-	+	-
119	64^R^	128^R^	256^R^	256^R^	0.125^S^	2^S^	-	-	+	-	-	+	-
120	64^R^	0.5^S^	0.25^S^	0.25^S^	0.125^S^	2^S^	-	-	+	-	-	+	-
133	64^R^	128^R^	256^R^	256^R^	0.125^S^	2^S^	-	-	+	+	-	+	-
134	64^R^	128^R^	256^R^	256^R^	0.125^S^	2^S^	-	-	-	-	-	+	-
Total no.							3	7	14	2	6	6	22

The antibiotic-resistant *S. aureus* indicated macrolide-, FA- or mupirocin-resistant isolates. Abbreviations for ERY, AZM, CLR, DTM, FA and MUP are the same as in Table 1. The macrolides, Fusidic acid and mupirocin resistance level were indicated in the MATERIALS AND METHODS ^R^, resistance; ^S^, susceptibility; ^H^, high level resistance; ^L^, low level resistance; +, positive; -, negative.

For cross-resistance profile in these isolates, we found the only FA-resistant (isolate no. 041) and 5 of 6 mupirocin-resistant isolates (except isolate no. 049) also belong to the 27 macrolide-resistant *S. aureus* isolates (Table 2). We further determined the resistance level of FA- and mupirocin-resistant isolates. The only FA-resistant isolate was identified to be low level resistance with a MIC of 2 μg/ml (Table 2). Among the 6 mupirocin-resistant *S. aureus* isolates, 1 (isolate no. 038, 16.7%) was low level resistance with a MIC of 256 μg/ml, and the other 5 (83.3%) were high level resistance with MICs of 512-1024 μg/ml (Table 2). These data demonstrated that macrolide-resistant *S. aureus* could be generally eradicated by FA and mupirocin, and there is an inclination for FA- and mupirocin-resistant isolates to develop multi-drug resistance.

6 MRSA were included in our collection and their resistance to macrolides, FA and mupirocin was also tested. For four macrolide antibiotics, our result showed all 6 MRSA isolates were resistant to erythromycin and 5 (83.3%) to other three macrolide antibiotics (Table 1 and 2). We found all 6 MRSA isolates were also susceptible to FA and mupirocin (Table 1 and 2), which suggested FA and mupirocin were still applicable to treat MRSA infections.

### Prevalence of antimicrobials resistance determinants

To determine the resistance genes or gene mutations in 28 antibiotic-resistant *S. aureus*, PCR was conducted (Supplementary Figure 1). We first detected macrolide-resistant genes in 27 macrolide-resistant isolates. The results showed that macrolide-resistant *S. aureus* isolates harbored *ermA* (11.1%), *ermB* (25.9%), *ermC* (51.9%), *erm33* (7.4%) as listed in Table 3. Additionally, of the 27 isolates, 1 (3.7%), 6 (22.2%) and 11 (40.7%) contained only *ermA*, *ermB* and *ermC*, respectively (Table 4). Finally, for resistant gene combinations including *ermA*+*ermC*, *ermA*+*erm33*, *ermB*+*ermC* and *ermC*+*erm33*, the ratios for the isolates each accounted for 3.7% (Table 4). These data manifested *ermC* was the major macrolide resistance determinant and multiple *erm* genes can be existed in one isolate. Meanwhile, there were 5 macrolide-resistant isolates which were found negative for *erm* and *msrA* genes (Table 2), revealing new unknown resistance determinants are responsible for the resistance of these isolates.

**Table 3:** Positive rates of resistant genes among 27 macrolide-resistant *S. aureus* isolates

Gene	*S. aureus*, % (n/27)	MSSA, % (n/21)	MRSA, % (n/6)
*ermA*	11.1% (3/27)	9.5 % (2/21)	16.7 % (1/6)
*ermB*	25.9% (7/27)	33.3 % (7/21)	0 % (0/6)
*ermC*	51.9% (14/27)	47.6 % (10/21)	66.7 % (4/6)
*erm33*	7.4% (2/27)	0 % (0/21)	33.3 % (2/6)
*msrA*	0 % (0/27)	0 % (0/21)	0 % (0/6)

**Table 4:** Distribution of macrolide resistance genes among 27 macrolide-resistant *S. aureus* isolates

Macrolide-resistant genes	No. of isolates (%)
*ermA* alone	1 (3.7%)
*ermB* alone	6 (22.2%)
*ermC* alone	11 (40.7%)
*ermA+ermC*	1 (3.7%)
*ermA+erm33*	1 (3.7%)
*ermB*+*ermC*	1 (3.7%)
*ermC*+*erm33*	1 (3.7%)

Among 21 macrolide-resistant MSSA isolates, 2 (9.5%), 7 (33.3%) and 10 (47.6%) contained *ermA*, *ermB* and *ermC*, respectively; while in 6 MRSA isolates, 1 (16.7%), 4 (66.7%) and 2 (33.3%) contained *ermA*, *ermC* and *erm33* (Table 2 and 3), respectively. Therefore, *ermC* was the predominant determinants in both MSSA and MRSA, and both *ermA* and *ermC* were more common among MRSA than MSSA. In addition, no *erm33* or *msrA* was found in MSSA; no *ermB* or *msrA* existed in MRSA.

The only FA-resistant isolate we identified belongs to low level resistance. Therefore, we detected related genes responsible for low level resistance [9, 10], but found no resistance determinants *fusB/C/D* genes or *fusE* gene mutations in this isolate. In addition, we also examined *fusA* mutations which are responsible for high level resistance [8]. Our result demonstrated that these mutations did not exist in our resistant isolate either. This suggests there may exist other unknown resistant mechanisms responsible for FA resistance in *S. aureus*.

For mupirocin-resistant determinants, the genes *mupA* and *mupB* were traditionally associated with high level mupirocin resistance. Therefore, we first examined the existence of these two genes. However, our PCR result showed that these genes were not found in the 5 high level resistant isolates, which suggested that other determinants, rather than *mupA* and *mupB*, contributed to the resistance. In addition, we also examined the low level resistance gene *ileS* in these high level resistance isolates, and found all were positive to *ileS* mutation A637G (Table 2), which manifested the *ileS* gene mutations might be associated with the high level mupirocin resistance. Next, for low level mupirocin resistance, we first detected in the only low level resistant isolate (No. 038, Table 2) the *ileS* mutations G1762T and G1891T, which are essential for the low level mupirocin resistance [15]. However, these two mutations were not found in the isolate. We further detected mutations including A637G and A2412T, which are not significantly involved in low level mupirocin resistance [15]. We found only A637G in the isolate (Table 2). These results suggested that A637G may play a role in low level mupirocin resistance as well as in high level mupirocin resistance.

In summary, we have detected the existence of macrolide- and mupirocin-resistant genes and gene mutations in 23 of 28 antibiotic resistant isolates. These 23 isolates were defined as resistant gene-positive *S. aureus*. No FA-resistant genes were found in the only resistant isolate. Within the other 5 antibiotic resistant isolates, we identified no known resistant genes or gene mutations.

### Molecular characteristics of resistant gene positive strains

**Figure 1:**
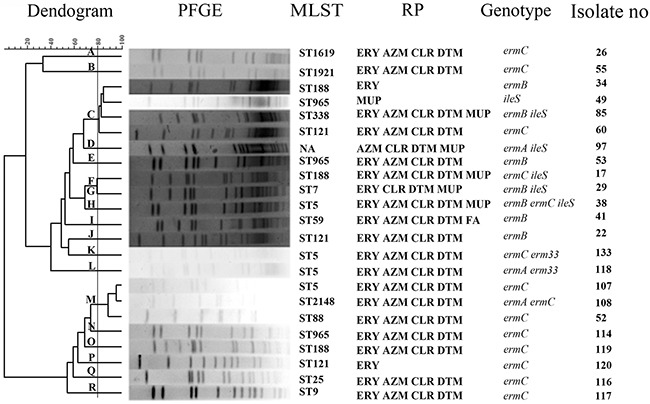
A dendrogram of MLST and PFGE The 23 genes-positive strains belonged to different 18 clusters from A to R. MLST, multilocus sequence typing; PFGE, pulsed field gel electrophoresis; RP, resistant phenotype; ERY, erythromycin; CLR, clarithromycin; AZM, azithromycin; DTM, dirithromycin; FA, fusidic acid; MUP, mupirocin; ST, sequence type; NA, not available.

To study the homology of resistant gene positive *S. aureus*, the 23 isolates were molecularly typed by multilocus sequence typing (MLST). Except 1 isolate (no. 97) whose sequence type (ST) is novel and being prepared for submission and identification, the MLST results of the remaining 22 isolates can be divided into 13 different STs as listed in Figure 1. Among these STs, ST5 which was the most common ST existed in four isolates. ST188, ST965 and ST121 together appeared in each of 3 isolates. The remaining 9 STs were belonged to 9 different strains. The 21 macrolide-resistant gene positive isolates had all 13 STs (Figure 1). 4 of these isolates which mostly harbored *ermC* (3/4) belonged to ST5, followed by 3 contained ST188 and ST121, and 2 contained ST965. The remaining isolates had only single and diverse ST of other types. In addition, the only FA-resistant isolate belonged to ST59. Mupirocin-resistant isolates had 6 STs, with ST5 in the only low level resistant isolate and the rest 5 in high level resistant isolates. Next, pulsed field gel electrophoresis (PFGE) was utilized to further identify the 23 resistant gene positive isolates. Our result showed that PFGE patterns of the genomic DNA of these isolates were classified into 18 clusters (Figure 1). The isolates no. 34, 49, 85, 60 belonged to one cluster, and no. 107, 108, 52 to another cluster. Other isolates revealed the other 16 distinct clusters. In brief, the present study showed the STs and PFGE patterns of 23 resistant gene positive isolates were sporadic and heterogeneous, thus the phenomenon demonstrated antibiotic-resistant isolates existed diverse genetic backgrounds.

## DISCUSSION

Erythromycin was discovered several decades ago, it was often used due to the excellent tissue penetration and good oral absorption. With the use of new available macrolide derivatives in the 1980s, this group of antibiotics remains an important class of drugs for the treatment of a variety of community and hospital infectious diseases caused by Gram-positive and Gram-negative bacteria [2, 4]. As the other commonly used effective antimicrobials, FA and mupirocin are often used in the treatment of *S. aureus* infections, too. However, the resistance to macrolides, FA and mupirocin are rapidly increasing around the world.

In this study, the resistance rate of erythromycin (73.5%, Table 1) was higher than that in Belgium (37.4%) [20], Iran (42%) [21] and India (51.7%) [22], but lower than that in Korean (77.5%) [23] and Brazil (95.2%) [24]. Interestingly, compared with two previous reports (97.3% in MSSA, 98% in MRSA and 59.1% in *S. aureus*) [25, 26] surveyed in China, our study indicated different geographical regions and bacteria sources harbored diverse but increasing erythromycin resistant ratio. Of note, the overuse of erythromycin, which is often prescribed for impetigo therapy in China [27], may be the primary reason why the high level of resistance happened. Moreover, besides erythromycin, resistance to all the other tested macrolide antibiotics among MSSA isolates was significantly lower than that of MRSA isolates (Table 1), which was similar with early report [28]. These results also demonstrated that *S. aureus* co-holding macrolide resistance and virulence determinants in the hospitals would be prevalence.

Several high level resistant genes, such as *erm* genes, have been reported in *S. aureus*. It is notable that the predominance of these genes (i.g. *ermA* and *ermC*) was variable in several countries [4, 24, 29–31]. Here in China we found that, among the 27 macrolide-resistant *S. aureus* isolates, *ermC* was the most prevalent resistance determinant and *ermA* was less prevalent (51.9% vs 11.1%, Table 3).

Of note, the co-existence of *ermA* and *ermC* was found in 3.7% (1/27) isolates (Table 4), which was similar with the result from the literature in European countries (3%) [29], but different from that in Turkey (37.5%) [32]. Furthermore, we examined MIC values against macrolides in this *S. aureus* isolate co-harboring *ermA* and *ermC*, but found they were all relatively low. For this unexpected result, we speculate that the expression of *erm* genes would be in the control through ribosome stalling or riboswitch, which are general ways for antibiotic induction resistance [33, 34].

As FA has been licensed for decades in many countries [5], FA-resistant *S. aureus* emerged gradually and the recent resistance rates ranged from 1.4% to 52.5% in European countries [35], 7% in Canada and Australia [36] and <10% in most Asia countries and the United States [5, 36]. Here, among 34 *S. aureus* isolates investigated, we found only 1 FA-resistant isolate (2.9%, Table 1) with low level resistance. The low resistance rate is consistent with others [37, 38], except a study from Wenzhou with the extraordinarily high resistance rate of 14.3% [39]. The low FA-resistance rate and MIC value may be due to the clinical practice available in China since 1999, and as a topical cream since 2003 [27].

The only FA-resistant isolate revealed resistance to four macrolides with MIC each of 256 μg/ml (Table 2). Comparing to the high resistance ratio (79.4%, 27/34) of macrolides, this result showed FA was more effective than macrolides, and suggested macrolides may be inapplicable to deal with FA-resistant *S. aureus*. The other *S. aureus* isolates including 6 MRSA were all susceptible to FA (Table 1), which was in accordance with previous report that MRSA were sensitive to FA [25].

Similarly, since mupirocin was first introduced into clinic in 1985, there has appeared mupirocin-resistant *S. aureus* isolates in 1987 [40]. Afterwards, it has been increasingly reported the mupirocin-resistant *S. aureus* in many countries, such as UK 0.3% [41], Spain 11.3% [42], USA 13.2% [43], Belgium 3.6% [44], India 6% [45], Greece 4.4% [46], Korea 5% [47], Turkey 45% [48]. Additionally, previous studies indicated that the mupirocin resistance in China was ranging from 0 to 6.6% [27, 49–51]. However, the mupirocin-resistant *S. aureus* in our study was 17.6% (6/34, Table 1). The reason for rapidly increased resistance to mupirocin and its high MIC value may be its extensive use in China in recent years [25].

For mupirocin-resistant determinants, we checked both high level resistance genes *mupA* and *mupB* and low level resistance *ileS* gene mutations in all 6 resistant isolates. However, No *mupA* or *mupB* was found in high level resistant isolates. We could only identified *ileS* mutation A637G in both low level and high level resistant isolates (Table 2), suggesting there might exist the possibility that high level resistant strains evolve from low level resistant ones bearing A637G mutation. We also found other *ileS* mutations other than A637G in the only low level resistant isolate, but the connection between these mutants and the resistant phenotype awaits more investigation.

For cross-resistance profile in the mupirocin resistant isolates, we found that 4 high and 1 low level mupirocin-resistant isolates were resistant to multiple macrolides (Table 2), which indicated high and low level mupirocin-resistant *S. aureus* had no much difference in macrolides resistance. Interesting, all MRSA isolates were susceptible to mupirocin (Table 1), which were similar to the result of a previous report [52].

The antibiogram of *S. aureus* in our study demonstrated high resistance rate of macrolides and low resistance rate of FA and mupirocin, indicating macrolides are not appropriate agents any longer to treat infections caused by *S.aureus*, and the other two antibiotics remain an effective treatment. In addition, there was a trend of the occurrence of multiple resistance among *S. aureus*, thus further restrictions on the use of these antimicrobials are demanded to curtail the spread of antibiotics resistant *S. aureus*. For resistant mechanisms of these three antibiotics in our study, *ermC* was the major resistant determinant for macrolides resistance, and A637G of *ileS* for both high and low level mupirocin resistances. Other unknown resistant determinants are responsible for FA resistance. At present, we are sequencing the genome of this intriguing isolate, hoping to find novel FA-resistant determinants.

Among the 13 STs from 22 resistant gene positive strains ST5 was the most prevalent ST (18.2%, Figure 1), which is similar to the result of a previous study [39]. It has been reported ST59 of community-associated MRSA is mostly observed in Asia and the United States [53], but only one MSSA isolate in our specimens harbored ST59. We also found 2 MSSA and 1 MRSA isolates with ST121, which also indicated ST121 are mostly reported in MSSA than MRSA [25]. Additionally, ST121 isolates (13.6%, 3/22) were more prevalent than ST59 (4.5%, 1/22, Figure 1), which was in accordance with another investigation [25]. As reported in some Asia countries, ST239 or ST5 were the major STs among MRSA isolates [54–57]. In our study, ST5, but not ST239, was found in our MRSA isolates, suggesting MRSA distribution has considerable heterogeneity from different geographic areas.

It is worth noting that the 23 resistant gene positive isolates belonged to 18 clusters (Figure 1) and isolates with the same ST were attributed to different clusters. The highest similarity of 2 isolates in one cluster was no higher than 95.58% and the dissimilarity of two clusters was no lower than 33.31%. Traditionally, the isolates with the same phenotype and genotype show the same PFGE groups. While in our study, the same phenotype and genotype in antibiotics-resistant strains displayed diverse PFGE types. This phenomenon has also been reported previously [11]. In addition, ST5 (1-4-1-4-12-1-10) differs from ST965 (1-4-1-4-119-1-10) only in locus *pta* with one nucleotide difference due to a single recombination event, but these two STs were assigned to different clusters. Meanwhile, ST965 was found to be different in seven loci from ST338 (19-23-15-48-19-20-15), but they were assigned to one cluster. These findings suggest adaptation to varying environmental conditions occurs through microevolutionary changes within a single isolate [58, 59]. Notably, the 23 isolates with the similar resistance phenotype and genotype belonged to various STs and clusters, revealing the genetic relatedness among these isolates was weak and there was a high genetic heterogeneity. This heterogeneity further confirmed that monitoring of these antibiotics resistance among *S. aureus* should be strengthened.

## MATERIALS AND METHODS

### Bacterial isolates

A total of 34 nonduplicate *S. aureus* isolates including 6 (MRSA) were collected from clinical specimens of Lishui Central Hospital in Zhejiang province, China, between February 2010 and November 2011. These specimens came from tissues including blood, urine, sputum, wound exudates and abscess. All the isolates were identified by detecting the existence of the housekeeping gene *arcc* [60].

### MRSA identification

To identify MRSA isolates, a simplex PCR was used for the detection of *mecA* (Table 5) [61]. PCR products were sequenced by TSINGKE (Chengdu, China) and then confirmed by aligning to sequence of *mecA* gene of MRSA with NCBI Nucleotide Blast.

**Table 5:** Primers used in this study

Gene	Primer	Primer sequence(5’ to 3’)	Amplicon size(bp)	Reference
*mecA*	*mecA-F* *mecA-R*	5’-TCCAGATTACAACTTCACCAGG-3’ 5’-CCACTTCATATCTTGTAACG-3’	162	[61]
*IleS1*	*IleS1*-F *Iles1*-R	5’-ATTTCCCAATGCGAGGTGGT-3’ 5’-CGTGACCTGGTGCTGTATGT-3’	954	This study
	*IleS2*-F *IleS2*-R	5’-TCTGGTTCATCACACCGTGG-3’ 5’-GCACGGTTCACATCATCACG-3’	842	This study
	*IleS3*-F *IleS3*-R	5’-ACGTGATGATGTGAACCGTG-3’ 5’-TTGTTGGCATCGTGGGCATA-3’	322	This study
*mupA*	*mupA*-F *mupA*-R	5’-CATTGGAAGATGAAATGCATACC-3’ 5’-CGCAGTCATTATCTTCACTGAG-3’	443	[18, 71]
	*mupA*-up *mupA*-dn	5’-TATATTATGCGATGGAAGGTTGG-3’ 5’-AATAAAATCAGCTGGAAAGTGTTG-3’	457	
*mupB*	*mupB*-F *mupB*-R	5’-CTAGAAGTCGATTTTGGAGTAG-3’ 5’-AGTGTCTAAAATGATAAGACGATC-3’	674	[18]
*fusA*	*fusA1-F* *fusA1-R*	5’-ACGATGGAAGATCGTTTAGC-3’ 5’-TGGTCAGCTTTAGATTTTGGC-3’	1510	This study
	*fusA2-F* *fusA2-R*	5’-AGGTACAATGACATCTGGTTC-3’ 5’-TCTCTCATGATAGTTTCTCACC-3’	1259	This study
*fusB*	*fusB-F* *fusB-R*	5’-ATTCAATCGGAAACCTATAATGATA-3’ 5’-TTATATATTTCCGATTTGATGCAAG-3’	292	[67]
*fusC*	*fusC-F* *fusC-R*	5’-TCTCGGACTTTATTACATCG-3’ 5’-TGAGAAAGAGTGATGTATCAG-3’	348	This study
*fusD*	*fusD-F* *fusD-R*	5’-AATTCGGTCAACGATCCC-3’ 5’-GCCATCATTGCCAGTACG-3’	465	[39]
*fusE*	*fusE-F* *fusE-R*	5’- TGTTGGTGGAGAAATTATCGC-3’ 5’- CCTGATAAGTTAGTACGAACACG-3’	696	This study
*ermA*	*ermA-F* *ermA-R*	5’-TCTAAAAAGCATGTAAAAGAA-3’ 5’-CTTCGATAGTTTATTAATATTAGT-3’	645	[66]
*ermB*	*ermB-F* *ermB-R*	5’-GAAAAGGATCTCAACCAAATA-3’ 5’-AGTAACGGTACTTAAATTGTTTAC-3’	639	[66]
*ermC*	*ermC-F* *ermC-R*	5’-TCAAAACATAATATAGATAAA-3’ 5’-GCTAATATTGTTTAAATCGTCAAT-3’	642	[66]
*erm33*	*erm33-F* *erm33-R*	5’-TCTGCAACGAGCTTTGGGTT-3’ 5’-TCAAAGCCTGTCGGAATTGGT-3’	239	This study
*msrA*	*msrA-F* *msrA-R*	5’-TCCAATCATTGCACAAAATC-3’ 5’-AATTCCCTCTATTTGGTGGT-3’	163	[4]

### Antimicrobial susceptibility testing

The MICs of 34 *S. aureus* to erythromycin, clarithromycin, azithromycin, dirithromycin, mupirocin (all from Sangon, Shanghai, China) and FA (Adamas-beta, China) were determined by agar dilution method, according to the 2010 guidelines provided by the Clinical and Laboratory Standards Institutes (CLSI), and *S. aureus* strains ATCC29213 for macrolides & fusidic acid and ATCC25923 for mupirocin were taken as control [25, 62]. The MICs value used to indicate erythromycin, clarithromycin, azithromycin and dirithromycin resistance are ≥ 8 μg/ml in accordance with CLSI. For FA resistance, MIC thresholds of 2 to 64 μg/ml indicate low level resistance, and ≥ 128 μg/ml high level resistance [63, 64]. For mupirocin resistance, low level resistance is from 8 to 256 μg/ml and high level mupirocin resistance ≥ 512 μg/ml [65].

### DNA extraction

For 28 antibiotic-resistant *S. aureus* strains, each was cultured overnight on a Mueller-Hinton agar plate at 37°C. Then, a single bacterial colony was suspended in 1 ml sterile LB medium, and subsequently incubated at 37°C with vigorous shaking for 6 hours, followed by centrifugation at 13000 × g for 2 minutes. Next, the culture media was discarded, and the pellet was mixed with 100 μl of Tris-HCl (200 mM, pH 8.0) and 2μl of lysostaphin (1 mg/100 μl, Sangon, Shanghai, China). Finally, each sample was heated at 37°C for 1 hour, followed by 95°C for 15 minutes, and centrifuged at 13000 × g for 2 minutes. Supernatant containing genomic DNA was collected and used as template for the PCR assays.

### PCR amplification and DNA sequencing

Antibiotic-resistant *S. aureus* strains were used by PCR to detect resistance determinants as listed in Table 5 [2, 4, 18, 36, 39, 47, 66, 67]. PCR was carried out according to previous studies [47, 66, 68]. Early reports on *fusA* and *ileS* gene mutations were also included in our study for PCR detection [14, 15, 64, 67–70]. In order to clearly ascertain the position of putative mutations in these two genes, we divided the entire *fusA* gene into *fusA1* and *fusA2* fragments, and *ileS* gene into *ileS1*, *ileS2* and *ileS3* fragments according to a previous report [11]. For *mupA* detection, two pairs of *mupA* primers were used in the PCR reaction (Table 5) [18, 71]. PCR products were sequenced by TSINGKE and then confirmed by sequence comparison to corresponding genes in the Nucleotide sequence database of NCBI.

### Molecular typing of resistant gene-positive strains

The genetic relatedness among 23 resistant gene-positive *S. aureus* strains was typed by MLST and PFGE. According to the MLST database (http://saureus.mlst.net), seven housekeeping genes (*arcc*, *aroe*, *glpf*, *gmk*, *pta*, *tpi* and *yqil*) were amplified and sequenced for the determination of ST. Novel ST of DNA sequence was verified and deposited in *Staphylococcus aureus* MLST Databases (https://pubmlst.org/saureus/). PFGE typing was conducted by a modification of the protocol previously described [72, 73]. The homology of these strains was analyzed with BioNumerics software (Applied Maths). Strains with >80% similarity were assigned to the same PFGE clusters [74].

## CONCLUSION

This study indicates macrolides, especially erythromycin, are not appropriate agents any longer to treat skin infections caused by *S. aureus*; the gene *ermC* was the predominant determinant for macrolides resistance among *S. aureus*; mupirocin and FA remain effective drug candidates for the eradication of *S. aureus*; A637G mutation in *ileS* gene may result in low and high level mupirocin resistance. Additionally, the heterogeneity among 23 resistant gene positive strains supports the tendency for the continued dissemination of macrolides, FA and mupirocin resistance in *S. aureus*, and thus suggests the resistance of *S. aureus* to these antimicrobials should be continuously supervised and adequate infection control measures against these antibiotics-resistant isolates should be established.

## SUPPLEMENTARY MATERIALS FIGURE


