# Supplementary material for: Dissemination of macrolides, fusidic acid and mupirocin resistance among Staphylococcus aureus clinical isolates
Source: Oncotarget. 2017 Jul 22;8(35):58086–97. doi: 10.18632/oncotarget.19491 (PMC5601635; doi:10.18632/oncotarget.19491)
Supplement: Supplementary file 1 [file oncotarget-08-58086-s001.pdf]

Dissemination of macrolides, fusidic acid and mupirocin resistance among *Staphylococcus aureus* clinical isolates

SUPPLEMENTARY MATERIALS

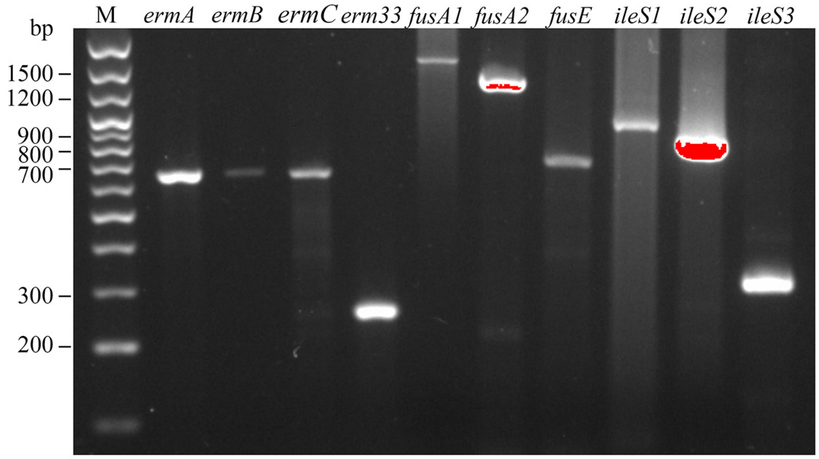

Supplementary Figure 1: The electrophoretogram of 7 resistant determinants of macrolides, fusidic acid and mupirocin. The *ermA*, *ermB*, *ermC* and *erm33* were belonged to macrolides; *fusA1*, *fusA2* and *fusE* to fusidic acid; and *ileS1*, *ileS2* and *ileS3* to mupirocin.
